# Evaluation of Autogenous Healing in Flexural Mortar Members by Chloride Ion Penetration Resistance

**DOI:** 10.3390/nano11061622

**Published:** 2021-06-21

**Authors:** Byoungsun Park, Youngcheol Choi

**Affiliations:** 1 Department of Environmental Systems Engineering, Sejong Campus, Korea University, Sejong-si 30019, Korea; bspark0927@korea.ac.kr; 2 Department of Civil and Environmental Engineering, Gachon University, Seongnam 13120, Korea

**Keywords:** autogenous healing, flexural member, chloride ion penetration test, mineral admixture, water flow test

## Abstract

In this study, we investigated the effects of mineral admixtures on the autogenous healing of flexural mortar members through a chloride ion penetration test. The mineral admixtures used were ground granulated blast-furnace slag (GGBS), fly ash, silica fume (SF), clinker binder, and clinker sand. Through a four-point bending test, a crack of approximately 100 μm was induced at the bottom of the flexural mortar member, and the chloride ion penetration depth through the crack was measured to evaluate the self-healing performance. Additionally, we analyzed the correlation between the self-healing performances, which was measured through water flow and water absorption tests. The experimental results showed that the chloride ion penetration depth decreased due to crack healing, and the self-healing performance of the GGBS and SF was the highest. It was found that the subtle change in the self-healing performance was more accurately evaluated by the chloride ion penetration test.

## 1. Introduction

Self-healing concrete has the property of being able to self-heal cracks through materials such as bacteria, capsules, and polymers that are added to the concrete [1,2,3]. Thus, self-healing concrete reduces maintenance costs and improves the durability of concrete structures, and has been actively studied recently [4]. Some researchers have also used mineral admixtures to conduct research on improving autogenous healing, which is a desirable unique characteristic of cementitious materials [5,6,7,8,9,10,11,12]. Autogenous healing is observed when cracks are healed due to further hydration of the unreacted binder and the precipitation mechanism of calcite. The mechanism of autogenous healing is different from that of self-healing induced by bacteria or encapsulated polymer [13]. 

Although the development of self-healing materials is an active research area, a standardized self-healing performance evaluation method has not been developed. Thus, various evaluation methods are currently used by different researchers [14]. Commonly used self-healing performance evaluation methods include the water flow and crack closing tests. The water flow test evaluates the self-healing performance by measuring the water flow through the crack via the condition of the hydrostatic head [15]. The water flow test is used to quantitatively evaluate the self-healing performance through a decrease in the water flow. However, a problem exists in that the water flow is measured differently even with the same surface crack width, depending on the flexure and shape of the crack [16]. Moreover, because a penetration-crack-induced specimen is used for the water flow test, it is difficult to evaluate the self-healing performance of the actual structure, as the cracks in both structures differ [17]. Unlike the water flow test, the crack closing test is used to measure the change in crack width on the surface of a specimen, using an optical microscope; however, it is unsuitable for investigating whether the inner crack has self-healed [18]. 

Therefore, various test methods have been proposed to evaluate self-healing performance [13,14]. Park and Choi investigated self-healing performance through a water absorption test based on ASTM C 1585 [13] and made comparisons with the results of the water flow test. Ahn et al. studied the applicability of various nondestructive testing methods (ultrasonic pulse velocity, surface-wave transmission, diffuse ultrasound, acoustic emission (AE), and coda wave interferometry) to evaluate the crack self-healing performance [19]. It has been reported that other nondestructive test methods, other than AE analysis, can be applied to all types of self-healing materials, and the AE analysis test method can be applied to the self-healing performance evaluation of a specimen mixed with capsules [19]. Tittelboom et al. investigated self-healing potential due to the further hydration of the pastes, in which supplementary cementitious materials (SCMs) are substituted by isothermal calorimetry [20]. It was reported that self-healing potential increased when ordinary Portland cement (OPC) was replaced by ground granulated blast-furnace slag (GGBS). 

Most of the proposed evaluation methods indirectly examine the filling ratio of cracks or the amount of self-healing material that can react when cracks occur. As self-healing concrete improves structural durability by preventing the penetration of harmful ions, it is necessary to investigate the change in the penetration depth of harmful ions due to self-healing; nevertheless, relevant research in this area is currently insufficient. Maes et al. examined the chloride ion penetration change due to the self-healing of cracked mortar specimens, but could not simulate the cracks in actual structures, because they used totally splitting crack induced specimens [21,22].

In this study, crack self-healing performance was evaluated based on the change in the chloride ion penetration depth due to the autogenous healing in flexural members. The self-healing performance of the cracked flexural mortar member was investigated through water absorption and chloride ion penetration tests. Additionally, a water flow test was performed using the totally splitting crack induced specimens to measure self-healing performance. The correlation between self-healing performance evaluated through each test method was analyzed.

## 2. Materials and Methods

### 2.1. Materials

In this study, OPC, GGBS, FA, SF, and clinker binder were used as binders. Their respective densities were 3.14 g/cm^3^, 2.9 g/cm^3^, 2.15 g/cm^3^, 2.35 g/cm^3^, and 3.16 g/cm^3^. The chemical composition of the raw materials was investigated using X-ray fluorescence (XRF) analysis, as shown in Table 1. For OPC and clinker, the phase composition was calculated using the Bogue equation, and their respective C_3_S, C_2_S, C_3_A, and C_4_AF were 57.7%, 16.8%, 7.0%, and 9.83% as well as 58.6%, 18.5%, 7.5%, and 9.0%, respectively.

**Figure 1:**
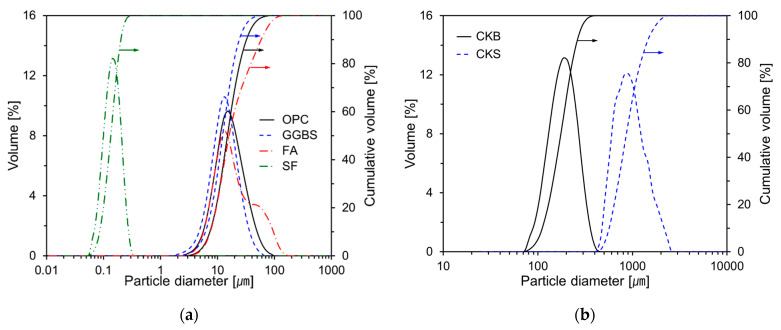
Particle size distributions of raw materials: (**a**) OPC, GGBS, FA, SF; (**b**) CKB, CKS.

Figure 1 illustrates the particle size distribution of the raw materials, which was measured using the laser diffraction method (Beckman Coulter LS 230, Brea, CA, USA). Figure 1a illustrates the particle size distribution of OPC, GGBS, FA, and SF, and their average particle sizes were 17.47, 13.16, 31.96, and 0.15 μm, respectively. Figure 1b illustrates the particle size distribution of the clinker binder and clinker sand. CKB and CKS represent the clinker binder and clinker sand, respectively. The clinker sand was manufactured to replace the fine aggregates when making the mortar specimens. The average particle sizes of the CKB and CKS were 213 and 950 μm, respectively.

In this study, polyvinyl alcohol (PVA) fibers were used to facilitate the induction of a certain crack width in the flexural specimen. RECS 15 (Kuraray, Tokyo, Japan), 8 mm long and 40 μm in diameter, was used as the PVA fiber.

### 2.2. Mixture Proportions

We prepared mortar specimens according to the mixing ratios shown in Table 2 to examine the effects of the SCMs and clinker mixture. The water–binder ratio was fixed at 0.3, and the weight ratio of the water, binder, and fine aggregate of the cement mortar was 0.3:1:1.5. The standard sand (ISS) and CKS specified in ISO 679 were used to manufacture the mortar specimens [23]. Approximately 30% and 60% of the GGBS were mixed with respect to the weight of OPC; 15% and 30% of the FA and 10% of the SF were also mixed. CKB (10%) was substituted with respect to the weight of OPC, and CKS was substituted at 5%, 10%, and 15% with respect to the weight of the fine aggregate. To ensure the workability of all formulations, approximately 0.5% of a poly-carboxylic chemical admixture was applied with respect to the weight of the binder. After mixing the binder for 30 s at a low speed through a planetary mixer, water was added and mixed for 1 min. Fine aggregates and a chemical admixture were then added and mixed for 2 min, and PVA fibers were slowly added and mixed for 1 min when an appropriate flow was obtained.

### 2.3. Mixture Proportions

#### 2.3.1. Compressive Strength

The mortar compressive strength was measured according to ISO 679 [23]. Mortar specimens for measuring compressive strength were prepared using a 40 mm × 40 mm × 160 mm mold. The prepared specimens were cured in a thermo-hygrostat chamber (temperature: 20 ± 1 °C, relative humidity (RH): >90%) for 24 h. Subsequently, they were cured in a water container at a temperature of 20 ± 1 °C for 28 days. The compressive strength of six specimens was measured, and the average was used as the resulting value.

#### 2.3.2. Water Absorption and Chloride Ion Penetration Tests

**Figure 2:**
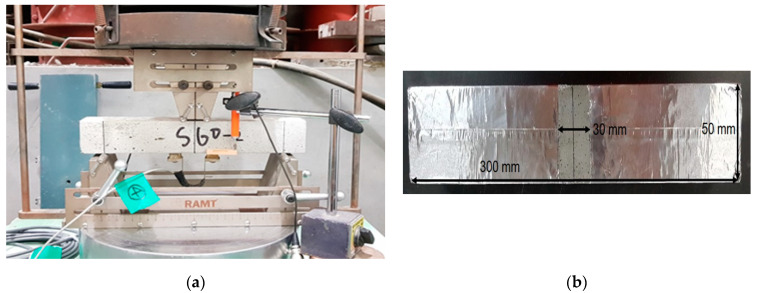
Preparation of cracked mortar member: (**a**) Four-point bending test; (**b**) Pretreatment of cracked specimens.

To evaluate the self-healing performance of the flexural cracks, a 50 mm × 50 mm × 300 mm flexural mortar specimen was prepared. The specimen was cured for 14 days in a chamber of 20 ± 1 °C and RH of ≥95%. The flexural crack in the center of the mortar specimen was induced through a four-point bending test, as shown in Figure 2a. The displacement of the center of the specimen and the crack width were measured according to the load through a linear variable differential transformer (LVDT) and a PI displacement transducer (PI-2-50, manufactured by TML, Tokyo, Japan). PI-2-50 has a gauge length of 50 mm and can measure a displacement of ±2 mm. After inducing, the crack at the lower end of the specimen was measured four times using an optical microscope, and the average value was used as the crack width. The crack width was set to approximately 100 μm when the load was unloaded. 

**Figure 3:**
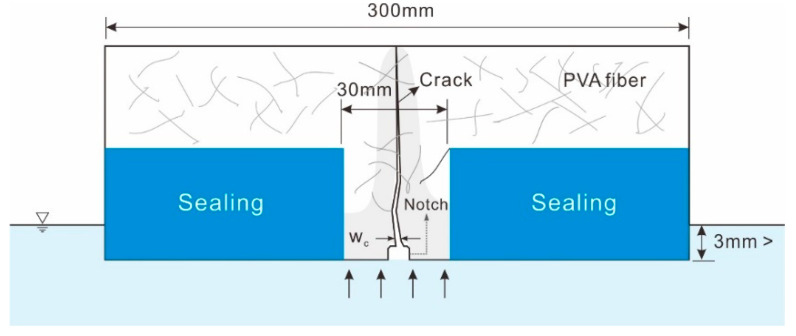
Diagram of water absorption test.

The rest of the cracked mortar specimens were sealed with aluminum tape, except for 30 mm around the crack at the bottom of the specimen, as shown in Figure 2b. This sealing was aimed at inspecting the change in absorption rate according to self-healing in the water absorption test. The specimen with aluminum tape was placed on a glass rod in a sealed container in a thermo-hygrostat chamber at a temperature of 23 ± 2 °C and RH of 60 ± 10%, when the age of the specimen was 14 days. Three millimeters of the lower end of the specimen was immersed in 0.2% calcium hydroxide (Ca(OH)_2_) aqueous solution for 56 days. To maintain the Ca(OH)_2_ concentration, the solution was replaced every week and stirred periodically. The absorption rate was measured using the method presented in ASTM C 1585 [24] and calculated by dividing the mass of the specimen that absorbed moisture by the area of the specimen and the density of the water. Three specimens were used per each variable. Figure 3 shows a diagram of the water flow test used in this study.

After the absorption rate test, the specimen was dried for 24 h in a dry chamber at a temperature of 40 °C. The dried mortar specimen was then placed on top of a glass rod in an acrylic sealed container in a thermo-hygrostat chamber at a temperature of 23 ± 2 °C and RH of 60 ± 10%. Three millimeters of the lower part of the specimen was immersed in NaCl solution having a concentration of 15% chloride ion for 28 days. The NaCl solution was replaced every week to maintain its concentration. After 28 days, the specimen was cut in the direction perpendicular to the crack, and a silver nitrate (AgNO_3_) solution was applied to measure the chloride ion penetration depth.

**Figure 4:**
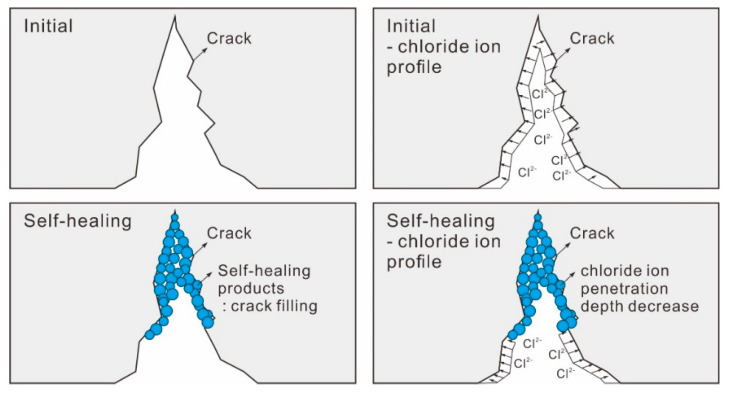
Cl penetration depth decreasing mechanism by crack self-healing.

Figure 4 shows the Cl penetration depth decreasing mechanism due to crack self-healing. A micro crack in the concrete structure acts as a pass to penetrate harmful ions such as chloride ion and sulfate ions. So, if concrete with micro cracks is immersed in chloride ion solution, chloride ion penetrates into the concrete through the micro crack. As can be seen in the figure, chloride ion penetrates to the top surface of the mortar specimens before crack healing. In the case of the cracked specimen after self-healing, the crack surface in contact with chloride ion solution is decreased, because the healing product fills the micro crack. Therefore, chloride ion penetration depth is decreased as shown in the figure. We evaluate the self-healing performance of the mortar specimen using this mechanism.

#### 2.3.3. Water Flow Test

In this study, the water flow test was used to evaluate the self-healing performance [13]. A cylindrical mortar specimen of diameter 100 mm × height 50 mm was used in the water flow test. The specimens were stored for 24 h in a thermo-hygrostat chamber at 20 ± 1 °C and RH ≥ 95%, then demolded and cured in a water tank at a temperature of 20 ± 1 °C for the first 14 days. After splitting the specimen into two pieces through the split tensile test method, a copper wire was placed in between to fix the crack width at 100 μm [13]. For the static water level test, a valve was installed at the lower end of, and at 250 mm above, the specimen. While maintaining a constant head, water was continuously supplied to measure the amount of water that passed through the crack in the specimen over time.

## 3. Results and Discussion

### 3.1. Compressive Strength

The compressive strengths of the specimens at 28 day are shown in Table 3. The strengths of Plain, S30, and S60 decreased as the mixing ratio of GGBS increased. The strengths of F15 and F30 also decreased, compared with Plain; however, the effect was insignificant. Compared with Plain, the compressive strengths of SF10 and CKB10 that used a clinker binder increased; there was no significant difference between the strengths of Plain and CKB10. CKS is a specimen in which the standard sand was substituted with clinker sand, and it had similar strength to that of Plain. Clinker sand, which has hydraulic properties, was expected to increase in strength because it generates hydrates. However, the effect of clinker sand on the compressive strength was insignificant.

### 3.2. Results of Water Absorption Test

**Figure 5:**
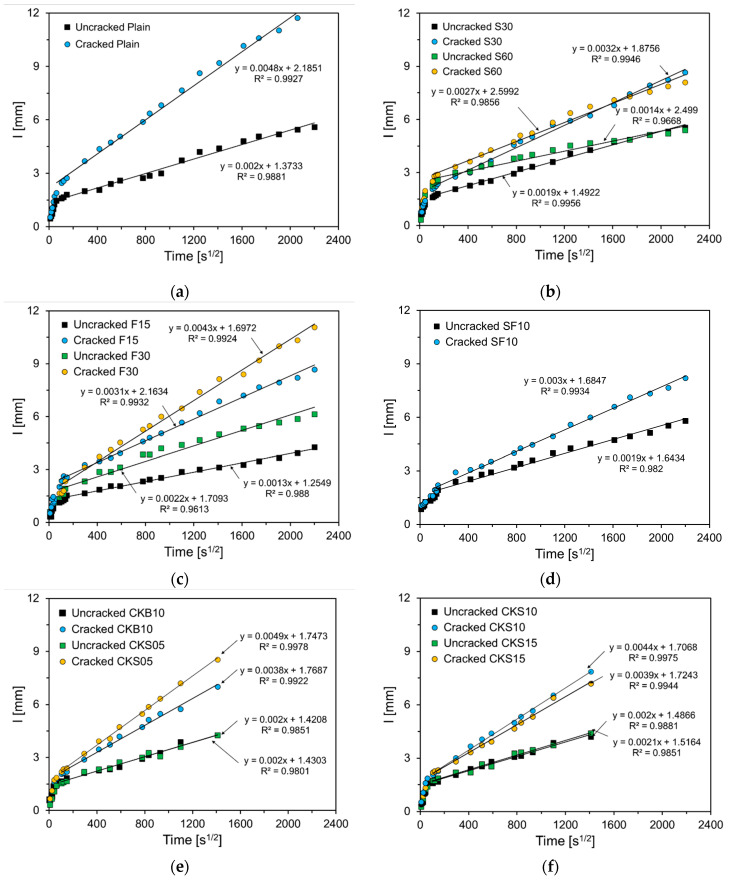
Water absorption test results: (**a**) Plain; (**b**) S30, S60; (**c**) F15, F30; (**d**) SF10; (**e**) CKB10, CKS05; (**f**) CKS10, CKS15.

The results of the water absorption test described in Section 2.3.2 are shown in Figure 5. The graphs depict the average absorption rates of the specimens composed of the first and second absorption slopes. The initial and secondary slopes are parameters influenced by the microstructure of cementitious materials. Therefore, the initial and secondary slope can be used to evaluate the connectivity of the pore network. The porous media absorbs moisture rapidly at the beginning, and then the rate of absorption slows down. The initial slope and the secondary slope represent the initial and late absorption rates, respectively. The water absorption rate of the cracked specimens increased, compared with that of the uncracked specimens. This is because the absorption area increased relative to the uncracked specimens, due to the crack surface. The water absorption indexes (Is) of the uncracked specimens at 56 days were similar, except for F15, which was smaller than those of the other specimens. The water absorption index of F30 was similar to that of Plain. 

Chahal et al. measured the absorption rate of concrete with FA substitution rates of 10%, 20%, and 30% [25]. The authors reported that the absorption rate was the lowest when the FA substitution rate was 10%, and the absorption rate was similar to that of Plain when the FA substitution rate was 30%. Foti et al. reported that the absorption rate decreased when substituting FA for OPC [26]. For the cracked specimens, the water absorption index of Plain was the highest at 11.8 mm, which was approximately 110% higher than that of the uncracked specimens. In S30, the water absorption indexes of the uncracked and cracked specimens were 5.7 mm and 9.3 mm, respectively, and the water absorption index of the uncracked specimens increased by approximately 63%. The water absorption indexes of S60 were 4.87 mm and 8.3 mm for uncracked and cracked specimens, respectively, and the water absorption index of cracked specimens rose by 70%. Additionally, the water absorption index of the F15 and F30 cracked specimens rose by 90% and 94%, respectively. For SF10, the water absorption index of the cracked specimen increased by 70%. When the clinker binder and clinker sand were used, the water absorption indexes of the uncracked specimens were similar regardless of the substitution of the clinker binder and sand. However, the water absorption index of the cracked specimens decreased with the substitution. The water absorption index of CKB10 was the lowest; in the case of CKS which used clinker sand, the water absorption index decreased as the amount of substitution increased.

**Figure 6:**
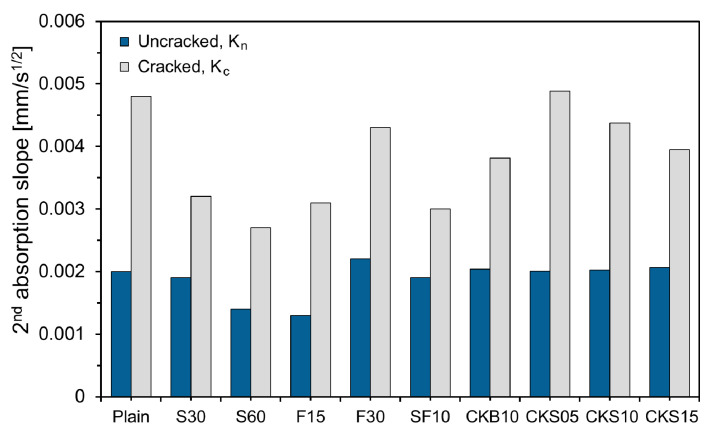
Second absorption slope of specimens.

Figure 6 depicts the second absorption slope of the specimens. As shown in the figure, the second absorption slope of the uncracked specimens was smaller than that of the cracked specimens. The difference between the cracked and uncracked specimens was largest in Plain and smallest in SF10. The difference in the second absorption slope between the cracked and uncracked specimens indicates the absorption rate through the crack; the smaller the difference, the smaller the crack area, due to self-healing [13]. Therefore, it can be seen that the specimen which substituted SCMs improved the self-healing performance compared to that of Plain. This is because the unreacted binders rose because of FA, GGBS, and SF, which hydrate slower than OPC, thus raising the self-healing products through further hydration [20]. The self-healing performance of S30 and S60 improved compared with that of Plain, and the increase in the substitution amount of GGBS showed no significant effect. The self-healing performance of F15 and F30 also improved compared with that of Plain; however, it was still less significant than that of GGBS. SF10 showed the largest difference in the second absorption slope, and the crack width reduction due to self-healing was the largest. Similar results have been reported in previous studies [7,20,27]. CKB10 exhibited an improved self-healing performance compared with that of Plain. Because the clinker binder has a larger diameter than OPC, the unreacted binder of CKB10 increased, unlike that of Plain, when cracking occurred.

Hence, it is concluded that the self-healing products increased, and the self-healing performance improved during further hydration. There was an improvement in the self-healing performance of CKS05, CKS10, and CKS15, which used clinker sand, and the healing performance improved as the clinker sand increased. Because the clinker sand has hydraulic properties, it has the same effect as the increase in the binder compared with Plain. Therefore, the self-healing products increased due to further hydration, thus improving the self-healing performance.

**Figure 7:**
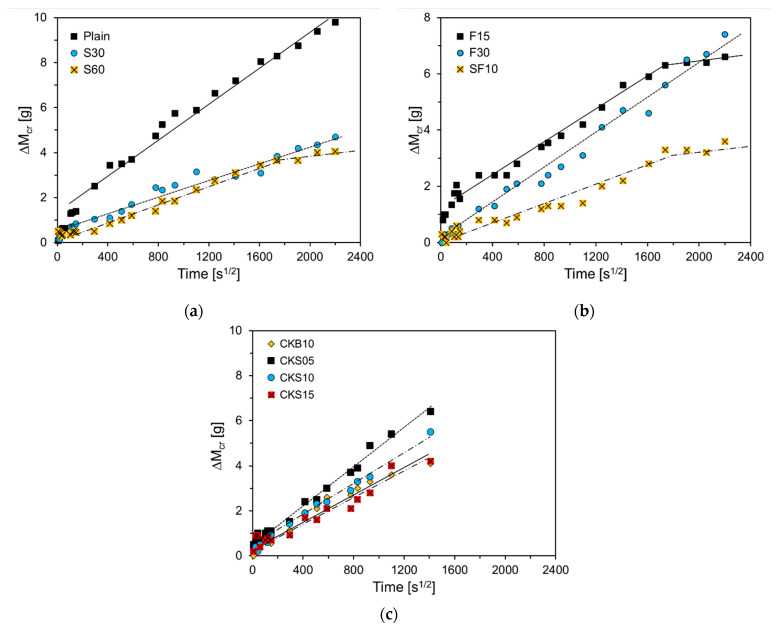
Difference in absorption rates between cracked and uncracked specimens: (**a**) Plain, S30, S60; (**b**) F15, F30, SF10; (**c**) CKB10, CKS05, CKS10, CKS15.

Figure 7 illustrates the difference in absorption rates between the cracked and uncracked specimens. Park and Choi evaluated self-healing performance by using the difference in absorption rates between cracked and uncracked specimens [13]. When the crack width is reduced by self-healing, water absorption through the crack decreases; when the crack is completely healed, the absorption rates of the uncracked and cracked specimens become identical. As observed in the graph, the slopes of S60, F15, and SF10 decreased on the 30th day. The zero difference in absorption rates between the cracked and uncracked specimens indicates that the crack was completely healed and there was no water absorption through the crack. Hence, it can be seen that S60, F15, and SF10 with reduced slopes hardly absorbed water through cracks from the 30th day. The experimental results showed that the self-healing performance of GGBS improved as the substitution amount increased, and the self-healing performance of FA declined as the substitution amount increased. For CKB10, CKS5, CKS10, and CKS15, which used clinker binder and sand, the absorption rate was measured until day 28, and no decline in the slope is observed.

### 3.3. Chloride Ion Penetration Test

**Figure 8:**
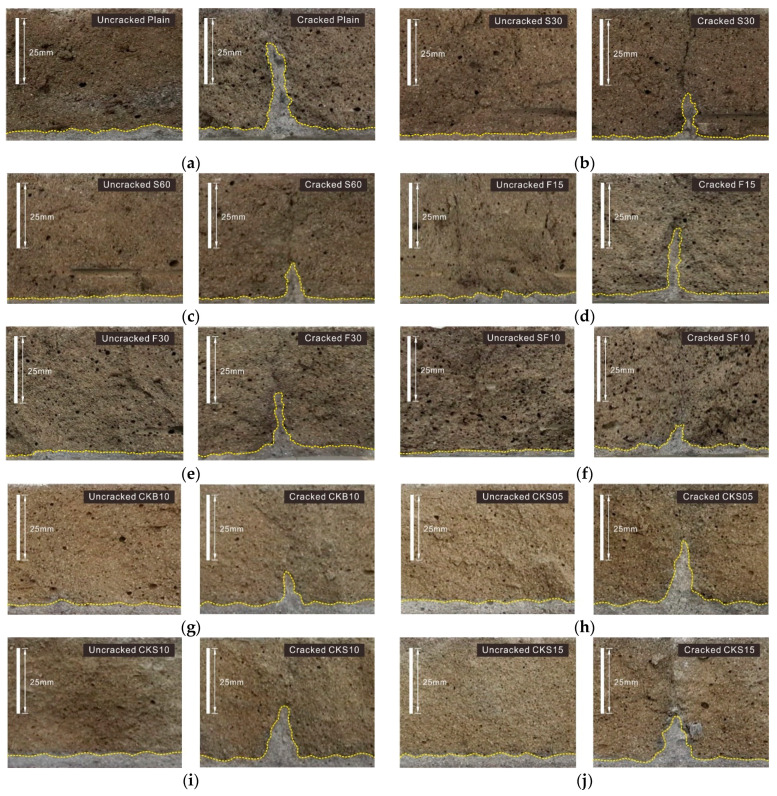
Chloride ion penetration profiles of specimens: (**a**) Plain; (**b**) S30; (**c**) S60; (**d**) F15; (**e**) F30; (**f**) SF10; (**g**) CKB10; (**h**) CKS05; (**i**) CKS10; (**j**) CKS15.

The chloride ion penetration profiles of the cracked and uncracked specimens are shown in Figure 8. The cracked specimens depicted in the figures refer to self-healed specimens that had been tested for water absorption. It can be observed in the figures that the penetration depth of the cracked specimens increased as chloride ions penetrated through the crack. The penetration depth of chloride ions in Plain was the largest, and the penetration depth decreased when SCMs, clinker binder, and clinker sand were substituted. This shows that when SCMs, clinker binder, and clinker sand were added, the crack width decreased due to self-healing.

The penetration depths of chloride ions measured in Figure 8 are summarized in Table 4. The chloride ion penetration depth of the uncracked specimens measured in Plain was 4.2 mm, and it decreased unlike when SCMs were added. In this study, a crack-induced specimen at 14 days was immersed in water for 56 days to investigate the self-healing performance of cracks by measuring water absorption rate. The test for measuring the penetration depth of chloride ions was performed using specimens at 70 days. According to the findings of a previous study, when 60%, 30%, and 10% of GGBS, FA, and SF were substituted, respectively, the resistance to penetration of chloride ions improved compared with Plain on the 70th day [28]. 

In the case of cracked specimens, the chloride ion penetration depth of Plain was the largest at 37.8 mm, and SF10 was the smallest at 11.9 mm. The self-healing performance of S60 was the best in the water flow and water absorption tests, whereas SF10 exhibited the best performance in the experiment for measuring the chlorine ion penetration depth. In S30 and S60, the penetration depth of chloride ion decreased as the substitution amount of GGBS increased. This is consistent with the results of water absorption tests. Because GGBS has a slower hydration reaction than OPC, the unreacted binders increase in long-term aged specimens in which GGBS is substituted [20,27]. Therefore, the penetration depth of chloride ions reduced by more than 20 mm, compared with Plain. As the substitution rate of FA increased in F15 and F30, the depth of chloride ion penetration decreased. This is different from the results of the water flow and water absorption tests. Chindasiriphan et al. reported that the effect of FA mixing rate on self-healing performance was insignificant [29]. On the other hand, Tittelboom et al. investigated the self-healing characteristics according to the FA substitution rate through isothermal calorimetry and reported that the self-healing potential was high when the FA substitution rate was 30% and 50% [20]. Therefore, the effect of the mixing rate of FA on the self-healing performance varied depending on the experiment. The self-healing performance of the GGBS-substituted specimen was higher than that of the FA-substituted specimen, which corresponds to the result of the water absorption test. In the case of CKS10, CKS05, CKS10, and CKS15, which used clinker binder and clinker sand, the chloride ion penetration depth of the uncracked specimens increased compared with Plain, whereas that of the cracked specimens decreased compared with Plain. The chloride ion penetration depth of CKB10 was the smallest at 15.4 mm, thus exhibiting the highest self-healing performance. The resistance to penetration of chloride ions due to self-healing improved as the substitution rate of clinker sand increased; however, it was insufficient compared with CKB10.

### 3.4. Results of Water Flow Test

**Figure 9:**
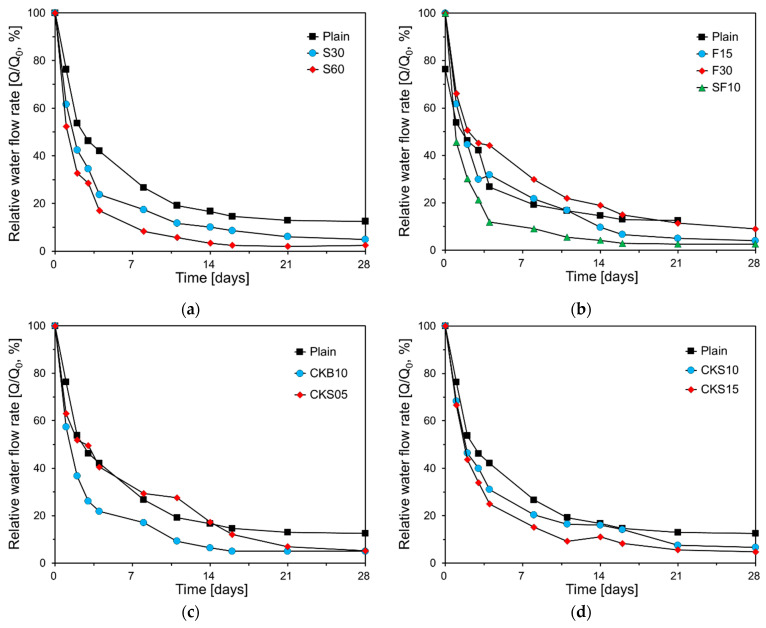
Water flow test results: (**a**) Plain, S30, S60; (**b**) Plain, F15, F30, SF10; (**c**) Plain, CKB10, CKS05; (**d**) Plain, CKS10, CKS15.

Figure 9 illustrates the results of the water flow test. The figure shows the average of three specimens. As seen in the figure, regardless of the specimen type, the water flow rate decreased as the self-healing period increased. The water flow rate decreased rapidly for the first seven days and slowly decreased thereafter. This is due to the mechanism of autogenous healing [13]. The reduction in the water flow of Plain was the smallest. The self-healing performance of GGBS improved as the substitution amount increased, and FA decreased as the substitution amount increased. Moreover, SF10 showed the highest water flow reduction rate along with S60. This is consistent with the self-healing performance evaluated through the absorption rate shown in Figure 7. Specimens that used clinker binder and clinker sand also had improved healing performance compared with Plain. Initially, the water flow reduction rates of CKB10 and CKS15 were high; however, those of CKB10, CKS05, CKS10, and CKS15 were similar at 28 days.

### 3.5. Comparison between the Results of Each Test Method

**Figure 10:**
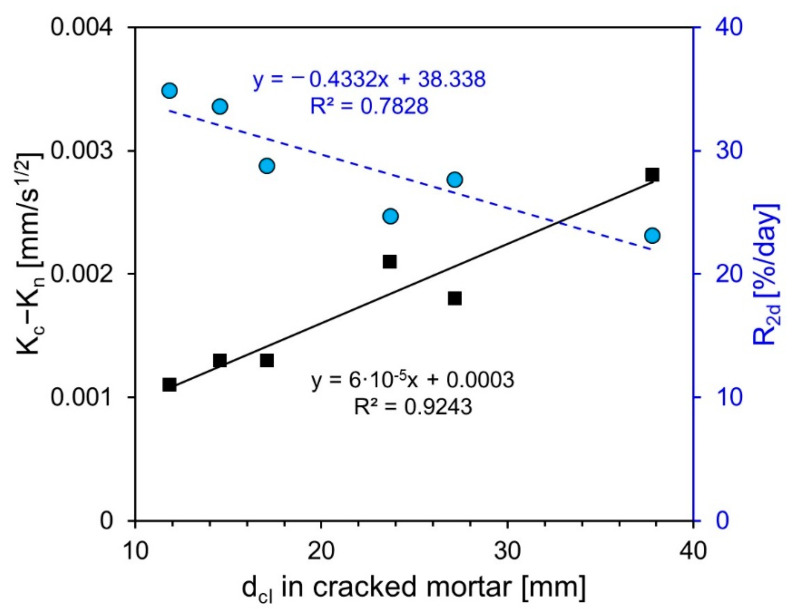
Correlation among self-healing performances of specimens including SCMs determined by each test method.

Figure 10 illustrates the correlation between the self-healing performance of the specimens, including SCMs, determined by each test method. In the graphs, K_c_ − K_n_ represents the difference in the second absorption slope between the cracked and uncracked specimens measured through the absorption rate test. d_cl_ represents the penetration depth of chloride ions in the self-healing specimen. R_2d_ of the y-axis represents the average water flow reduction in the first two days. While K_c_ − K_n_ and dcl had a high correlation with the determination coefficient of 0.9243, they were not linearly proportional depending on the specimen. In the case of F15 and F30, K_c_ − K_n_ and d_cl_ were not proportional. The self-healing performance of cementitious materials mixed with FA had varying results among different researchers [20,29]. The determination coefficients of dcl and R_2d_ were 0.7828, which was lower than that of K_c_ − K_n_ and d_cl_. However, it was found that R_2d_ increased as d_cl_ decreased, except for F15.

**Figure 11:**
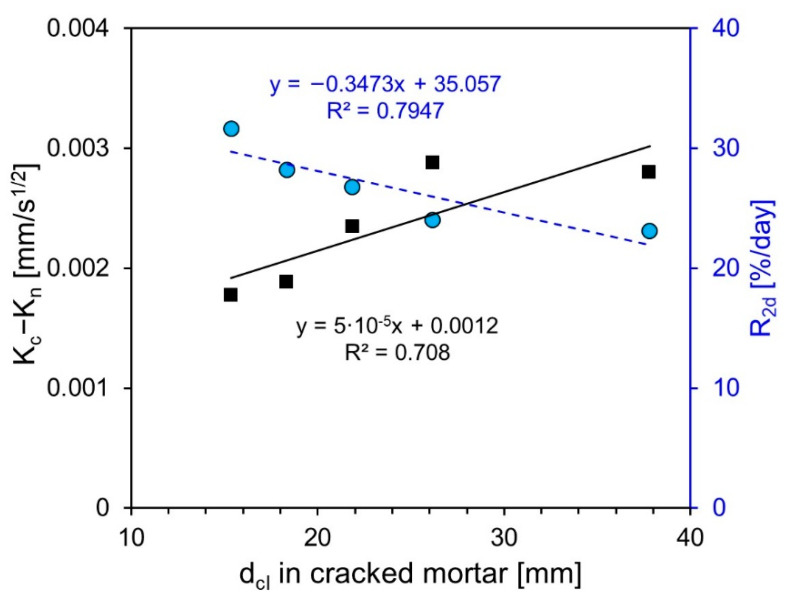
Correlation among self-healing performance of specimens including clinker binder and clinker sand determined by each test method.

The correlation between the self-healing performance of specimens (including clinker binder and clinker sand) determined by each test method are shown in Figure 11. The graph depicts a low correlation of 0.708 between K_c_ − K_n_ and d_cl_. In the case of the self-healing performance measured through the chloride ion penetration test, CKS05 and CKS10 increased compared with Plain but decreased or maintained the same level when measured through the water absorption test. Meanwhile, dcl and R_2d_ showed a linear relationship. In all test methods, the self-healing performance of CKB10 in which the clinker binder was substituted was the highest, and the clinker sand-specimen showed a proportional relationship between the substitution rate and self-healing performance.

As shown in Figure 10 and Figure 11, the chloride ion penetration test showed a clear difference even when the improvement in self-healing performance was small compared with other test methods. The self-healing performance of CKB05 was evaluated at similar levels to that of Plain in the water absorption and water flow tests. However, the chloride ion penetration depth decreased by approximately 30% in the chloride ion penetration test. In terms of the water flow test, the crack width was constant from the top to the bottom of the specimen because the self-healing performance was evaluated through penetration cracks. Therefore, there were few self-healing products precipitated inside the crack when the self-healing performance was low, making the change in water flow through the crack trivial. Thus, the self-healing performance of CKB05 was similar to that of Plain. In terms of the water absorption test, the second secondary slope was affected not only by crack healing, but also by the binder and aggregate. Hence, it is difficult to distinguish a slight improvement in the self-healing performance through the difference in the second secondary slope. Additionally, the water absorption test does not compare the difference in absorption rate before and after self-healing; rather, it measures the change in absorption amount during self-healing after crack induction, thus making it difficult to distinguish the time when self-healing occurs.

As the chloride ion penetration test evaluated the self-healing performance through flexural cracks, the crack widths of the upper and lower parts of the specimen varied. The crack width became narrower toward the top of the specimen, resulting in a micro crack. Moreover, the penetration depth decreased even with a slight improvement in the self-healing performance as it healed the narrowed micro cracks. Hence, unlike in the water flow test, the chloride ion penetration test revealed that the self-healing performance of CKB05 improved significantly compared with that of Plain. Furthermore, the crack width reduction due to self-healing was accurately measured relative to the water absorption test because the chloride ion penetration test was performed after inducing self-healing by supplying water.

## 4. Conclusions 

In this study, the effect of mineral admixture on the autogenous healing of flexural mortar members was investigated using the chloride ion penetration test. The conclusions can be summarized as follows:

1. Except for SF10 and S60, the compressive strength of the other specimens was similar to that of Plain.
2. In terms of cracked specimens, the second absorption slope of the SCM-substituted specimens and clinker-substituted specimens decreased compared with that of Plain.
3. When the chloride ion penetration depth of the uncracked specimens was measured, the SCM-added specimens decreased compared with Plain, and the specimens that included clinker binder and clinker sand increased compared with Plain. In contrast, in the case of cracked specimens, the chloride ion penetration depth of the specimens that included SCMs, clinker binder, and clinker sand decreased compared with Plain.
4. The results of the water flow test showed that the self-healing performance of SCM-substituted specimens increased more than that of Plain. When clinker binder and clinker sand were used, self-healing performance improved compared with Plain; however, the effect of the substitution rate was not observed.
5. Based on the correlation analysis between the test methods, it was found that the better the self-healing performance of the specimen, the higher the correlation between the test methods. Additionally, the chloride ion penetration test showed a clear difference even when the improvement in self-healing performance was small compared with other test methods.

## Figures and Tables

**Table 1:** Chemical compositions of raw materials.

	Chemical Compositions (wt.%)							
	CaO	SiO_2_	Al_2_O_3_	Fe_2_O_3_	MgO	K_2_O	Na_2_O	SO_3_
OPC	63.13	21.05	4.71	3.23	3.06	1.67	0.17	1.05
GGBS	45.20	29.30	13.80	0.53	4.13	0.45	0.28	3.59
FA	5.24	52.4	24.30	6.96	1.46	1.56	0.98	2.15
SF	0.17	91.76	0.39	0.90	1.23	0.97	0.77	0.41
Clinker	64.34	22.87	4.96	2.94	1.28	0.82	0.23	0.44

**Table 2:** Mixture proportions of mortar specimens.

Labels	W/B (-)	Binder (g)					Sand (g)		PVA Fiber (wt.% by Binder)
		OPC	GGBS	FA	SF	CKB	ISS	CKS	
Plain	0.3	100	-	-	-	-	150.0	-	0.5
S30		70	30	-	-	-			
S60		40	60	-	-	-			
F15		85	-	15	-	-			
F30		70	-	30	-	-			
S10		90	-	-	10	-			
CKB10		90	-	-	-	10	150.0	-	
CKS05		100	-	-	-	-	142.5	7.5	
CKS10		100	-	-	-	-	135.0	15.0	
CKS15		100	-	-	-	-	127.5	22.5	

**Table 3:** Compressive strength of specimens at 28 days.

Labels	Plain	S30	S60	F15	F30	SF10	CKB10	CKS05	CKS10	CKS15
Compressive strength (MPa)	53.3	51.0	42.6	52.3	51.5	57.8	54.9	53.2	53.9	51.8

**Table 4:** Chloride ion penetration depth of the specimens.

Penetration Depth (mm)	Plain	S30	S60	F15	F30	SF10	CKB10	CKS05	CKS10	CKS15
Uncracked, d_n_	4.2	2.4	2.5	3.3	2.7	2.1	4.3	4.8	4.7	4.5
Cracked, d_c_	37.8	17.1	14.6	27.2	23.7	11.9	15.4	26.2	21.9	18.3
d_c_/d_n_ (-)	8.9	7.2	5.9	8.3	8.9	5.6	3.6	5.4	4.6	4.1

## Data Availability

Data sharing not applicable.
